# Rapid Shifts in Bacterial Community Assembly under Static and Dynamic Hydration Conditions in Porous Media

**DOI:** 10.1128/AEM.02057-19

**Published:** 2019-12-13

**Authors:** Hannah Kleyer, Robin Tecon, Dani Or

**Affiliations:** a Soil and Terrestrial Environmental Physics, Department of Environmental Systems Science, Swiss Federal Institute of Technology in Zurich (ETH Zürich), Zürich, Switzerland; Chinese Academy of Sciences

**Keywords:** microbial ecology, bacterial community assembly, absolute quantification, quantitative real-time PCR (qPCR), microbial interactions, Fluidigm, porous media

## Abstract

The complexity of natural soils presents a challenge to the systematic identification and disentanglement of governing processes that shape natural bacterial communities. Studies have highlighted the critical role of the soil aqueous phase in shaping interactions among soil bacterial communities. To quantify and improve the attributability of soil aqueous-phase effects, we introduced a synthetic and traceable bacterial community to simple porous microcosms and subjected the community to constant or dynamic hydration conditions. The results were expressed in terms of absolute abundance and show species-specific responses to hydration and nutrient conditions. Hydration dynamics exerted a significant influence on the fraction of less-abundant species, especially after extended incubation periods. Phylogenetic relationships did not explain the group of most abundant species. The ability to quantify species-level dynamics in a bacterial community offers an important step toward deciphering the links between community composition and functions in dynamic terrestrial environments.

**IMPORTANCE** The composition and activity of soil bacteria are central to various ecosystem services and soil biogeochemical cycles. A key factor for soil bacterial activity is soil hydration, which is in a constant state of change due to rainfall, drainage, plant water uptake, and evaporation. These dynamic changes in soil hydration state affect the structure and function of soil bacterial communities in complex ways often unobservable in natural soil. We designed an experimental system that retains the salient features of hydrated soil yet enables systematic evaluation of changes in a representative bacterial community in response to cycles of wetting and drying. The study shows that hydration cycles affect community abundance, yet most changes in composition occur with the less-abundant species (while the successful ones remain dominant). This research offers a new path for an improved understanding of bacterial community assembly in natural environments, including bacterial growth, maintenance, and death, with a special focus on the role of hydrological factors.

## Abstract (precis)

The composition and activity of soil bacteria are central to various ecosystem services and soil biogeochemical cycles. A key factor for soil bacterial activity is soil hydration, which is in a constant state of change due to rainfall, drainage, plant water uptake, and evaporation. These dynamic changes in soil hydration state affect the structure and function of soil bacterial communities in complex ways often unobservable in natural soil. We designed an experimental system that retains the salient features of hydrated soil yet enables systematic evaluation of changes in a representative bacterial community in response to cycles of wetting and drying. The study shows that hydration cycles affect community abundance, yet most changes in composition occur with the less-abundant species (while the successful ones remain dominant). This research offers a new path for an improved understanding of bacterial community assembly in natural environments, including bacterial growth, maintenance, and death, with a special focus on the role of hydrological factors.

## INTRODUCTION

The mechanisms by which soil hydration dynamics affect microbial community composition and function remain poorly understood due to the complexity of natural soil-microbe systems. In their natural environments, bacterial communities respond to dynamic changes in hydration status by rapid changes in community composition and functioning, such as following a rainfall event in desert soil (1) or after simulated dry-wet cycles of soil cores (2–4). The response to changes in hydration occur quickly (i.e., the “Birch effect”), within hours (2, 5), with different response groups for rewetting events observed (5) and legacy response groups following prolonged dry periods (4). Studies have shown that the state of and variations in the soil aqueous phase control the diffusion of soluble nutrients, microbial respiration, and species dispersal and interactions (6–8). Indeed, evidence from field and laboratory studies has demonstrated that long- and short-term variations in soil water content can alter the structure of soil bacterial and fungal communities (1, 4, 9–14). The community response remains difficult to predict and quantify due to the inherent complexity of soil environments, with numerous factors jointly affecting bacterial communities during rewetting events. An avenue for disentangling the controlling factors is to reduce the complexity of the system and manage environmental variables that could affect community composition (15–18) in settings that mimic natural systems.

Here, we present a simplified system of porous microcosms (composed of glass beads) that form three-dimensional microbial habitats enabling accurate control of hydration status and other physicochemical factors. We have used a synthetic community composed of 11 bacterial species (19) that belong to common soil phyla (*Proteobacteria*, *Actinobacteria*, and *Firmicutes*) (Table 1). In terms of functional traits relevant to soil environments, members in the community contribute to carbon cycling and maintenance of soil fertility. Specifically, the selected species include nitrogen fixers (Rhizobium etli, Xanthobacter autotrophicus, and Pseudomonas stutzeri), a denitrifier (Pseudomonas stutzeri), a biocontrol agent (Pseudomonas protegens), degraders of organic contaminants (Xanthobacter autotrophicus, Burkholderia xenovorans, and Arthrobacter chlorophenolicus), and species able to enter dormancy with or without spore formation (Micrococcus luteus, Streptomyces violaceoruber, Paenibacillus sabinae, and Bacillus subtilis). Escherichia coli was also added as a nonsoilborne model species. The wide phylogenetic range of the community encompasses different life strategies that could provide insights into expected responses in natural soil bacterial communities. We subjected the community to wet-dry cycles and constant (prescribed) hydration conditions. The study enables species-level detection of changes in community composition and separation of effects on the dominant species and less-abundant species that are rapidly established in the microcosm. The primary objective of this study was to quantify effects of hydration dynamics in unsaturated porous microcosms on the composition of a synthetic bacterial community and contrast these with the effects of prescribed static hydration conditions. We posited that drier conditions, with reduced and spatially disconnected aqueous microhabitats, would decrease interspecies competitive interactions and thus foster coexistence and promote diversity (20–22). Additionally, we evaluated the potential effects of nutrient levels on bacterial community response to hydration conditions by considering high and low nutrient levels as an independent experimental factor. We seek to gain new insight into causal links between bacterial community assembly and dynamic hydration conditions to better understand behavior in unsaturated natural habitats found in most soils, where thin aqueous films constrain cell motility and limit nutrient diffusion pathways (8, 23). We aim specifically to elucidate the role of the soil aqueous phase, a key environmental factor that links climate, hydrology, and environmental microbiology with different land use and biomes (24, 25).

**TABLE 1:** Bacterial species composing the synthetic bacterial community^*a*^

Species	Strain	Phylum or class	Origin	Cell type	Motile	Spore formation
Arthrobacter chlorophenolicus	A6^T^	*Actinobacteria*	Soil	Rods/cocci	+/−	−
Streptomyces violaceoruber	A3(2)	*Actinobacteria*	Soil	Filaments	−	+
Micrococcus luteus	DSM 20030^T^	*Actinobacteria*	Soil	Cocci	−	−
Bacillus subtilis	168 *trp*^+T^	*Firmicutes*	Soil	Rods	+	+
Paenibacillus sabinae	T27^T^	*Firmicutes*	Rhizosphere	Rods	+	+
Rhizobium etli	CFN 42^T^	*Alphaproteobacteria*	Rhizosphere	Rods	+	−
Xanthobacter autotrophicus	7C^T^	*Alphaproteobacteria*	Soil	Rods	−	−
Burkholderia xenovorans	LB400^T^	*Betaproteobacteria*	Rhizosphere	Rods	+	−
Pseudomonas protegens	CHA0^T^	*Gammaproteobacteria*	Rhizosphere	Rods	+	−
Pseudomonas stutzeri	CMT.9.A	*Gammaproteobacteria*	Rhizosphere	Rods	+	−
Escherichia coli	MG1655	*Gammaproteobacteria*	Human gut	Rods	+	−

a We selected strains that are well characterized at the genomic and phenotypic level and that span a wide diversity of bacterial phyla. All strains grow aerobically and can be cultivated under standard laboratory conditions. All species are commonly found in soil, and all were originally isolated from soil or rhizosphere environments (with the exception of E. coli, used here as a nonsoilborne outsider species). A superscript T indicates a type strain.

## RESULTS

### Community convergence under a range of nutrient and water conditions.

**FIG 1:**
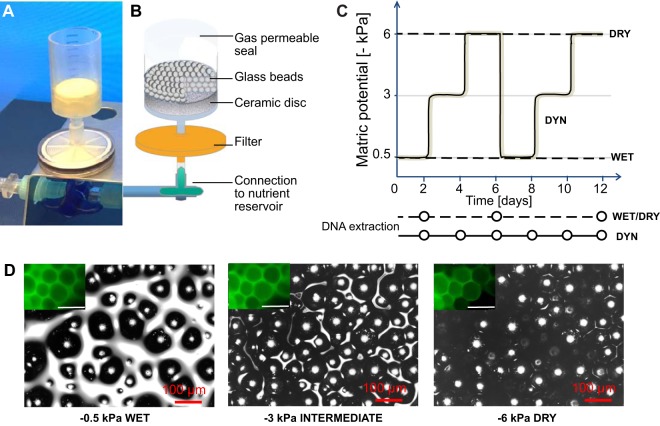
Experiments in hydration-controlled microcosms. (A and B) Photograph (A) and illustration (B) of the microcosms used in this study. Glass beads formed a porous habitat that could be colonized by the bacteria. The level of water saturation in the porous bacterial habitat was varied by applying defined suction to the system (expressed as matric potential value in kilopascals; see Materials and Methods for details). (C) Diagram of the experimental scheme contrasting dynamic (DYN) and constant (control) hydration regimes with values ranging from near water saturation (WET, −0.5 kPa) to relatively drier (DRY, −6 kPa). Each hydration regime was tested with low and high nutrient concentrations (0.01× and 0.1× TSBM liquid medium, respectively). (D) Micrographs depict the water configuration on the surface of a glass bead microcosm at the three levels of water saturation used in our experiments. Grayscale images were obtained with scanning laser microscopy (reflected light), while fluorescent images (insets; scale bar = 100 μm) were obtained with epifluorescence microscopy following the addition of fluorescein to the water (green coloration).

**FIG 2:**
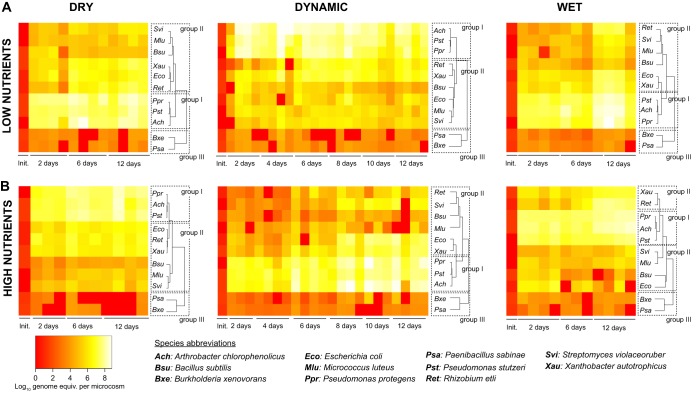
Bacterial species growth in microcosms. (A and B) The synthetic bacterial community was exposed to dynamic drying-rewetting cycles and compared to static wet or dry conditions under low-nutrient (A) or high-nutrient (B) conditions (0.01× and 0.1× TSBM liquid growth medium, respectively) for a maximal period of 12 days after inoculation. Heatmaps show the species absolute abundances (log transformed) calculated based on the absolute number of genome equivalents (equiv.) detected in each microcosm. The initial inoculum (Init.) and 4 independent replicate microcosms per treatment and time point are shown (when only three replicates are shown, it indicates that the fourth replicate produced insufficient amounts of DNA for analysis). Individual species were clustered based on the Bray-Curtis dissimilarity index. We defined three species response groups based on the results of hierarchical clustering across treatments, as follows: group I comprises the species that were consistently present at a high abundance level (up to 10^8^ genome equivalents per microcosm within 12 days); group II comprises species that had intermediate levels of abundance and whose growth response to treatments was varied; finally, group III comprises species that consistently showed very low abundance levels.

**FIG 3:**
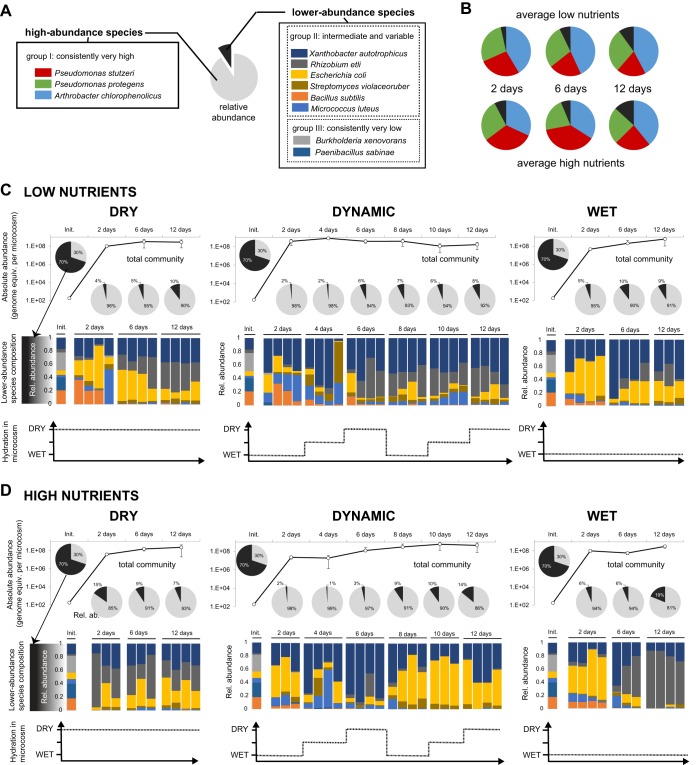
Variations in bacterial community composition. (A) Species in the synthetic bacterial community grouped based on their growth response in microcosms. (B) Pie charts show the relative abundances of individual species of the group I (all other species are pooled in the lower-abundance fraction). Values for different hydration treatments were averaged. Colors are as in panel A. (C and D) Details of community growth and composition of exposed DRY, WET, and DYN conditions with 0.01× TSBM (C) or 0.1× TSBM (D). Absolute community abundance is the sum of each species' number of genome equivalents per microcosm. Error bars represent one standard deviation (SD). Histograms show the composition of the lower-abundance subset of the community (consisting of species from groups II and III). The initial inoculum (Init.) and 3 or 4 independent replicate microcosms per treatment and time point are shown.

We first assessed the growth and structure of the synthetic community of bacterial species (Table 1) under contrasted nutrient and hydration regimes in microcosms mimicking soil (Fig. 1). Initially, each bacterial species was present at (approximately) equal biomass concentrations. The porous microcosms were subsequently incubated at constant temperature and under different hydration regimes over a period of 12 days (Fig. 1C). We expected that the range of hydration conditions applied would result in large differences in the aqueous-phase configurations at the microscale level relevant to bacterial life and interactions (Fig. 1D). Species abundance was expressed as the number of genome equivalents (≈individual cell numbers) calculated from quantitative real-time PCR (qPCR) amplification of genomic DNA (see Materials and Methods; see also Fig. S1 and S2 in the supplemental material). The largest fraction of bacterial growth occurred within the first 2 days postinoculation (Fig. 2 and 3). After 12 days, the total estimated number of cells was within a similar order of magnitude (≈10^8^ per microcosm, or ≈2.6 × 10^8^ per g glass beads) in all treatments (Fig. 3), suggesting that the system had reached its carrying capacity. Irrespective of hydration and nutrient conditions, the bacterial community composition drifted from its initial and relatively even proportions of all species to an assembly dominated by the same three species (Fig. 2 and 3). Community composition patterns were relatively similar across the various treatments and were largely reproducible between different sampled microcosms (Fig. 2 and S3). Moreover, after 12 days of incubation, there was no significant difference in community evenness (scored by Shannon’s equitability index) across the different treatments (Fig. S3).

### Clustering of bacterial species into growth response groups.

The analysis of absolute abundances showed that all members of the bacterial community grew in the microcosms, albeit reaching very different numbers of genome equivalents spanning 5 orders of magnitude (10^3^ to 10^8^) (Fig. 2). The species were clustered based on Bray-Curtis dissimilarity for each combination of hydration regime and nutrient concentration (Fig. 2). The results showed that *A. chlorophenolicus*, P. protegens, and P. stutzeri were always grouped together (group I), with individual species consistently reaching 10^6^ to 10^7^ cells per microcosm within 2 days and 10^7^ to 10^8^ cells within 12 days. A second group (II), composed of E. coli, *R. etli*, *X. autotrophicus*, *S. violaceoruber*, M. luteus, and B. subtilis, exhibited intermediate abundance levels (10^4^ to 10^6^) that gradually increased toward the end of the incubation period (up to 10^6^ to 10^7^). Species in this group did not represent a single cluster; instead, clustering organization varied across treatments (Fig. 2). Finally, *P. sabinae* and *B. xenovorans* formed a third group (III) that consistently clustered together in the analysis. Both species only grew to 10^3^ to 10^5^ cells, and that number remained somewhat stable over time. The observed grouping was clearly distinct from clustering based on phylogeny (Fig. S4).

Over time and across treatments, the high-abundance species of group I represented 81 to 98% (high-nutrient conditions) and 90 to 98% (low-nutrient conditions) of the community composition (Fig. 3). The dynamics of the abundance of group I relative to the rest of the community (groups II and III, comprising all lower-abundance species) exhibited a similar pattern, attaining dominance within the first 2 days after inoculation, followed by a slow increase in lower-abundance species over time (Fig. 3; one exception being microcosms under high-nutrient and dry conditions, where the dominance of group I increased over time). Given that species of group III only represented 0.003 to 0.03%, the variation in community composition was mostly driven by species of group II (2 to 19%). Indeed, only species belonging to group II showed significant variations in absolute and relative abundances as a function of hydration, nutrients, or time (Fig. 2 and 3). B. subtilis was persistently detected 2 days after inoculation; however, its relative abundance faded over time in all microcosms. Despite their low abundance, *S. violaceoruber* and M. luteus remained detectable at all sampling times. After 12 days, the most abundant species in group II were E. coli, *R. etli*, and *X. autotrophicus*, although composition patterns varied across treatments. Some temporal patterns emerged, especially under high-nutrient conditions, as follows: the absolute numbers of E. coli decreased over time under wet conditions compared to dynamic or dry conditions (Fig. 2), although after 12 days, the difference was significant only compared to dry conditions (*P* = 0.01 with a two-tailed *t* test on 4 replicates). Reciprocally, *R. etli* became more abundant in wet microcosms than in dynamic or dry microcosms (*P* = 0.002 and 0.003, respectively). As a result, E. coli and *R. etli* showed mirror profiles of relative abundance under wet conditions, which was explained by the combined effect of an increasing *R. etli* population and the growth arrest and population reduction in E. coli (Fig. S5). Conversely, E. coli increased in relative abundance under dynamic hydration and high-nutrient conditions, and it did so by growing relatively faster than the rest of the community, especially during the second cycle of hydration (Fig. 2). A similar second cycle pattern was observed with *S. violaceoruber* and *X. autotrophicus* under a dynamic hydration regime (Fig. 2 and 3). Notably, the filamentous bacterium *S. violaceoruber* reached >1% relative abundance in the dynamic treatment after 8 to 12 days of incubation (Fig. 3 and S3), and thus, its growth appeared to be stimulated by the dynamic hydration regime.

### Individual species fitness.

**FIG 4:**
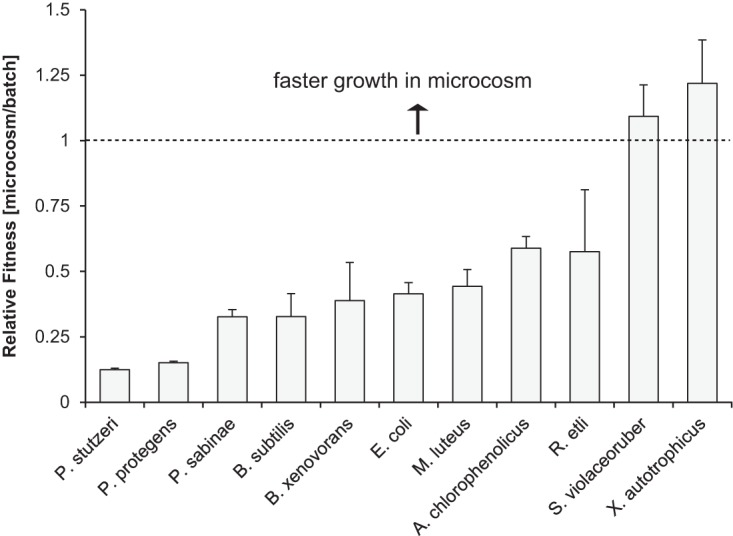
Comparisons of growth in microcosms and batch cultures. Relative fitness is expressed as the ratio of generation time measured in microcosm (after 2 days) and shaken liquid (batch) culture with high nutrient concentration (see Table 2). Values shown are averages for the DRY, WET, and DYN microcosms (error bar = 1 SD). All species grew faster in batch than in glass bead microcosms (ratio, <1), with the exception of *S. violaceoruber* and *X. autotrophicus*.

We examined whether the reproductive success of bacterial species in a microcosm could be predicted from their growth kinetics measured individually in shaken batch cultures using the same growth medium (Fig. S6). With a doubling time of 0.3 h, P. protegens and P. stutzeri were the fastest-growing species in shaken liquid media, followed by E. coli, M. luteus, B. subtilis, *A. chlorophenolicus*, *R. etli*, *P. sabinae*, *B. xenovorans*, *S. violaceoruber*, and *X. autotrophicus* (Table 2). These results matched the dominance of the two *Pseudomonas* spp. observed in microcosms. However, neither the high abundance of *Arthrobacter* spp. nor the exact rank of lower-abundance species matched the results from liquid cultures. We compared the average doubling times of individual species measured in porous microcosms after 2 days (and in the presence of the other species) to the shortest doubling time measured for each species in batch cultures. The ratio of these values, defined here as relative fitness, varied importantly among species in the community (Fig. 4 and S7). Although P. protegens and P. stutzeri showed the largest decrease in fitness among community members, they nevertheless had high reproductive success in microcosms. At the other end of the relative fitness landscape, and unlike all other species in the community, *S. violaceoruber* and *X. autotrophicus* grew faster in the microcosms than in batch cultures (Fig. 4), suggesting a positive effect of unsaturated conditions and heterogeneous porous structures on the fitness of these two species.

**TABLE 2:** Generation times in microcosms and individual species grown in batch culture^*a*^

Species group	Hydration conditions	Generation time (h) by species										
		Ach	Svi	Mlu	Bsu	Psa	Ret	Xau	Bxe	Ppr	Pst	Eco
Microcosms after 2 days	DRY	2.6	2.4	3.4	5.1	7.4	2.5	3.1	ND	2.1	2.5	2.8
	WET	2.3	2.4	2.6	3.3	6.3	3.1	3.0	5.1	2.0	2.4	2.6
	DYN	2.7	2.9	3.0	5.0	7.1	6.1	3.9	8.7	2.1	2.6	3.2
Batch cultures		1.5	2.8	1.3	1.4	2.3	1.9	4.0	2.5	0.3	0.3	1. 2

a The average generation time of each species growing in microcosm was calculated for high-nutrient conditions after 2 days based on the number of genome equivalents estimated from qPCR. We compare these values to the shortest generation times measured for species grown individually in batch cultures under the same nutrient conditions and during the exponential phase of growth. Ach, *Arthrobacter chlorophenolicus*; Bsu, *Bacillus subtilis*; Bxe, *Burkholderia xenovorans*; Eco, *E. coli*; Mlu, *Micrococcus luteus*; Ppr, *Pseudomonas protegens*; Psa, *Paenibacillus sabinae*; Pst, *Pseudomonas stutzeri*; Ret, *Rhizobium etli*; Svi, *Streptomyces violaceoruber*; Xau, *Xanthobacter autotrophicus*.

## DISCUSSION

The processes that govern changes in soil microbial community composition in natural environments are inherently complex and difficult to decipher due to numerous interactions that are neither easy to measure nor controllable (26). To overcome some of these methodological limitations, we have used a simplified synthetic bacterial community grown in elementary hydration-controlled porous microcosms (Fig. 1). The relatively short duration of the experiments (12 days) was patterned after standard changes in rainfall events in temperate regions or subsequent drying following a rainfall event on a desert soil (1). Our primary interest was in the effects of dynamic drying and rewetting regimes on bacterial community assembly relative to prescribed static hydration conditions in unsaturated porous microcosms (mimicking soil habitats). The bacterial species in the synthetic community (10 soil bacteria plus E. coli) were phylogenetically, morphologically, and physiologically diverse (Table 1), with doubling times ranging from 20 min to 4 h for species grown independently in shaken liquid cultures (Table 2 and Fig. S6). One of the novel aspects of the approach presented here is the ability to quantify absolute abundances of community members grown in porous media under known hydration and nutrient levels. The ability to track bacterial members under dynamic hydration conditions could pave the way for establishing causal principles that affect community organization in soil and other complex habitats.

### Role of hydration regimes.

Surprisingly, total bacterial community growth was somewhat unaffected by the regimes (despite very different aqueous environments), reaching in each case high cell numbers per microcosm (Fig. 2 and 3). Similarly, the growth of bacterial species that showed consistently very high abundance (group I) and very low abundance (group III) was not significantly different under various hydration regimes. In contrast, the growth of species from group II varied in absolute or relative abundance after repeated 6-day hydration cycles while other parameters were kept constant (Fig. 2 and 3). Most notably, under high nutrient concentrations, E. coli, *R. etli*, *S. violaceoruber*, and *X. autotrophicus* demonstrated unique growth behavior that translated into changes in community composition as a function of hydration regimes (Fig. 3). An important observation was that the community composition induced by dynamic hydration did not represent an average of wet- and dry-associated patterns. Instead, dynamic hydration conditions have shown unique characteristics, such as the relatively higher proportions of *S. violaceoruber* and E. coli at the end of the incubation period. Moreover, *S. violaceoruber* was the only species that showed a positive growth response specific to the dynamic regime. The strongest growth response was observed in *R. etli*, which was highly stimulated by a constantly wet environment. Overall, these results supported our hypothesis that community patterns would differ under contrasted hydration regimes, albeit in our experiments, only a subset of the bacterial community was demonstrably affected. As a result, lower-abundance species were better indicators of the impact of hydration regimes on community composition in microcosms than were high-abundance species. Although our observations were based on a simplified bacterial system, these results were consistent with observations that low-abundance bacterial taxa disproportionately contribute to community dynamics in many ecosystems (27, 28) and with a global meta-analysis of soil bacterial communities suggesting that rarer bacterial taxa are important determinants as well as good predictors of community structure (29).

A key yet challenging step is to link these hydration effects to specific physical, physiological, and ecological processes, including hydric stress, nutrient diffusion, and interspecies competition. With respect to ecological interactions, our prediction that drier conditions (associated with smaller and disconnected aquatic habitats for bacteria) would reduce competition and result in higher community diversity (Shannon’s equitability) in the microcosms was not supported by the results (Fig. 3 and S3). Nevertheless, our data hinted at putative competitive interactions that could be further explored, such as the replacement of E. coli by *R. etli* over time under constantly wet conditions (Fig. 3 and S5), or the general outcompeting of B. subtilis and M. luteus by the rest of the community (Fig. 3). As to nutrient availability and water stress, we speculate that despite the onset of aqueous-phase fragmentation for the drier conditions, residual liquid connectivity and constant supply of labile carbon and nutrients (notably, amino acids, glucose, and mannitol) at the lower boundary were sufficient to support community growth in all treatments, suggesting that the conditions in our microcosms were less selective than are natural soils. This could explain why E. coli, a human gut commensal that typically does not thrive in soil but instead declines over time (30, 31), showed high abundance thanks to short doubling times (Table 2 and Fig. S6). Steeper changes in water content (e.g., complete drying of the microcosms prior to rewetting) may be needed to generate more marked effects on community composition. So-called “drought legacy effects” have indeed been observed in column experiments using natural soil communities (4).

### Unique bacterial assembly in porous media.

The global community pattern, dominated by a trio of species (Fig. 3 and S3), was very similar and temporally stable across a range of moisture and nutrient conditions, even though in our simplified system, these conditions created contrasted environments (Fig. 1). Our results suggested an overarching effect of unsaturated porous habitats on the organization of the dominant fraction of the community, thus adding to the selective role of nutrient sources (32) and interspecies interactions (33) in structuring bacterial communities. Similarly, in a previous study, we showed that the assembly of a synthetic bacterial community in a shaken culture differs from its assembly in porous media (19). Our conclusions are further supported by recent studies demonstrating the profound effects that porous and water-unsaturated environments can exert on bacterial gene expression (34) or community functions (21).

The reproductive success of individual bacterial species in the synthetic community varied in hydrated microcosms (Fig. 2). Hierarchical clustering of species based on absolute abundances (Fig. 2) differed markedly from groupings based on phylogenetic distances (Table 1 and Fig. S4), the origin of bacterial strains (soil or rhizosphere), or their cell morphology and motility (Table 1). Although the high abundance of *Pseudomonas* spp. in our microcosms could correctly be inferred from high specific growth rates measured in liquid culture medium, the prevalence of *Arthrobacter* spp. in all microcosms defied predictions based on growth kinetics (Table 2 and Fig. S6). The dominance of pseudomonads is in line with observations that soil gammaproteobacteria grow faster and use more diverse resources than do other bacteria (35), and with shorter generation times (Table 2). For that reason, gammaproteobacteria in general and pseudomonads in particular are considered copiotrophs (36) and are classified as efficient competitors (12). Conversely, *Arthrobacter* bacteria have been referred to as a model for oligotrophic lifestyle (37) that have evolved to be successful colonizers of surface-attached soil habitats (8) in the presence of a highly mixed microbial concourse (38). The fitness of lower-abundance species in microcosms was similarly not accurately predicted by individual growth rates in liquid (Table 2 and Fig. 4). In particular, *S. violaceoruber* and *X. autotrophicus* proved to be on average fitter in hydrated porous media than in shaken batch cultures (Fig. 4). In sum, our results delineated a unique bacterial assembly in hydrated microcosms, which (so far) defies simple predictions from known data.

### Value of resolving absolute abundance.

An important aim of our research was to disentangle the mixed processes of growth, maintenance, and death that lead to the observed community patterns. This was made possible by the quantification of absolute abundance at the species level, which is typically not available in metagenomic studies of natural soil bacterial communities. In our study, the estimation of absolute cell counts was instrumental in detecting the more subtle effects associated with hydration regimes. In addition, the detected number of genome equivalents (Fig. 2) demonstrated that all species grew to some extent in the microcosms, even though some of them (group III) were rapidly outcompeted and were not apparent in relative abundance analyses (Fig. 3). The value of such an approach is illustrated in Fig. S5, focusing on a subset of species that showed temporal changes in relative abundance patterns. In essence, it helped us identify the correct mechanistic processes involved in the formation of specific patterns of community composition among multiple scenarios of equal explanatory power. This analysis showed that in our experiments, a majority of the compositional patterns could be explained by differential growth rates between species, while the (perhaps more intuitive) mechanism of growth versus decline (i.e., one species increases in number while concomitantly another species stalls or dies) was much rarer in our data set. Of course, the prevalence of one mechanism over the other may have been a direct consequence of the simplified system used in this study and may not be the rule in natural soil systems. A limitation of DNA-based assessment of microbial community composition (based on 16S rRNA gene) is the inability to discriminate live, dead, and dormant cells, which could lead to false-positive results. The use of propidium monoazide, which penetrates only membrane-compromised cells and inhibits PCR amplification, could help detect only living bacteria in the community (39).

In conclusion, the use of a synthetic bacterial community in combination with simplified unsaturated soil-like microcosms permitted a systematic exploration of certain hydration-controlled principles of community assembly and composition. We highlighted the overarching role of physical factors in generating specific diversity patterns, and we pointed at the prominent response of the lower-abundance community members to changes in environmental conditions. We argue that spatiotemporal heterogeneity is yet another essential component, together with bacterial taxonomic and functional diversity, which we should not overlook as we advance ecological theories of bacterial community assembly.

## MATERIALS AND METHODS

### Synthetic bacterial community and growth conditions.

The bacterial strains assembled in the synthetic community used in this study are listed in Table 1. All strains are available from the Leibniz-Institute German Collection of Microorganisms (DSMZ), as follows: A6 (DSM 12829), 168 (DSM 402), LB400 (DSM 17367), MG1655 (DSM 18039), DSM 20030, T27 (DSM 17841), CHA0 (DSM 19095), CMT.9.A (DSM 4166), CFN 42 (DSM 11541), A3(2) (DSM 40783), and 7C (DSM 432). All strains were routinely grown on tryptic soy broth (TSB; VWR International, Leuven, Belgium) supplemented with 1% mannitol (TSBM). TSB is a complex (rich) medium that is used to grow a wide variety of aerobic and facultative anaerobic bacteria. It contains enzymatic digests of casein and soybean (amino acids) and glucose. We added mannitol as an extra carbon source to sustain bacterial growth, especially that of Rhizobium etli. To assemble the community, each strain was first grown individually on 0.1× TSBM agar plates for 48 h at room temperature. Then, bacterial strains were harvested with a sterile cell spreader from the plates and resuspended in 0.01× TSBM in individual test tubes. Subsequently, each individual suspension was diluted in 0.01× TSBM in order to obtain an optical density at 600 nm (OD_600_) value of 0.1. Equal volumes of each species suspension were mixed together in a single tube and then diluted 1,000 times in 0.01× TSBM. This diluted mixed suspension of all species served as the inoculum in microcosm experiments, as detailed below. For growth experiments in batch culture, bacterial strains were recovered from 0.1× TSBM agar plates prepared in same way as described above and suspended in 0.1× TSBM liquid medium (high-nutrient conditions) or 0.01× TSBM medium (low-nutrient conditions). After adjusting the OD to 0.2, each individual pure culture was transferred in four replicates to a multiwell plate at a final optical density of 0.1 and culture volume of 220 μl. Growth was followed over 37 h at room temperature in a microplate reader (Spark microplate reader; Tecan, Maennedorf, Switzerland) by measuring the OD every 5 min, with cells mixed prior to each measurement by linear shaking for 10 s.

### Microscopy to visualize liquid film.

Water containing fluorescein, in combination with glass bead microcosms, was visualized with a DM6000 epifluorescence microscope (Leica Microsystems, Heerbrugg, Switzerland) using a 10×/0.30 HC PL Fluotar objective or a 40×/0.60 CORR HCX PL Fluotar L dry objective. Fluorescein was used at a final concentration of 0.1%. Grayscale fluorescence images were recorded with a DFC350 FX camera (Leica Microsystems) using an L5 filter cube for fluorescein (exciter, 480/40 nm; emitter, 527/30 nm; beamsplitter, 505 nm). The LAS acquisition software (Leica Microsystems) was used for image analysis. Image brightness was optimized with LAS according to sample type and filter cube with a Gamma function set to 1. Bead surface and aqueous film images were obtained with a VK-X200 three-dimensional (3D) laser scanning microscope (Keyence International, Mechelen, Belgium) using the VK Viewer acquisition software (Keyence International) and a 20× objective.

### Experiments in hydration-controlled porous microcosms.

The hydrated porous microcosms (Fig. 1A and B) were prepared as follows. We placed a ceramic disc at the bottom of cut 20-ml plastic syringes (Henke Sass Wolf, Tutlingen, Germany) 33 mm in height. The porous ceramic disc, with a diameter of 18.4 mm and thickness of 8.5 mm, was cut from a 10^5^-Pa porous plate (Soilmoisture Equipment Corp., Santa Barbara, CA, USA) and glued (and sealed) at the bottom of a syringe compartment. The ceramic material was selected with pores sufficiently small (<1 μm) to prevent the passage of bacterial cells to the liquid reservoir. The assembled system was immersed in ultrapure water (Thermo Fisher Scientific, Waltham, MA, USA) and sterilized by autoclaving (121°C, 10^5^ Pa) for 20 min, followed by incubation in a vacuum chamber for 1 h in order to remove any remaining air bubble. The next preparation steps were performed in a microbiological safety cabinet and with sterile material to prevent contamination. Each microcosm was mounted on a Filtropur S 0.45-μm syringe filter (Sarstedt, Nuembrecht, Germany) plugged in a three-way valve (B. Braun, Melsungen, Germany). The valves were connected serially with up to 12 (syringe) units hydrated via a common liquid nutrient reservoir (a 500-ml bottle containing 250 ml of 0.1× TSBM or 0.01× TSBM for high or low nutrient concentrations, respectively) via a 100-cm Heidelberger extension line (B. Braun). To create a porous environment for the bacterial community, we added 0.38 ± 0.02 g of sterile glass beads with diameter of 80 to 120 μm on top of the porous disc within the syringe compartment.

The microcosm hydration status (i.e., the water potential) was controlled by adjusting the height of a liquid column measured by the vertical distance between the bead surface and the free liquid medium surface in the reservoir. By lowering the reservoir bottle relative to the microcosm, we induced suction that partially withdrew liquid from the porous microcosm. The suction mimics effects of matric potential (*ψ_m_*) in draining or drying soil as related to capillary and adsorptive forces (41–43). The matric potential is expressed as energy per unit volume of water or as a force per unit surface area (i.e., units of suction of negative pressure [−kPa]). The suction (negative pressure) prescribed to a microcosm is calculated using the equation *ψ_m_* = ρ*gh*, where ρ is the density of water, *g* is the acceleration of gravity, and *h* is the height of the liquid column (42). For example, a 20-cm liquid column (below the surface of glass beads in the microcosm) mimics a matric potential of ≈ −2 kPa. Hence, lower *ψ_m_* values induce drier conditions in the microcosms (relative to full saturation). To allow gas exchange but prevent evaporation, we sealed each microcosm with Parafilm (with a small hole for pressure equilibration). Prior to bacterial inoculation, all microcosms were first saturated, set at a matric potential of −0.5 kPa (corresponding to nearly liquid-saturated conditions), and then kept for 24 h under sterile conditions (to ensure that all excess liquid was drained from the microcosms). Bacterial strains were mixed as a community as described above, and each microcosm was inoculated with 10 μl of a bacterial community suspension (0.01× TSBM containing each species in equal proportions based on OD measurements) by pipetting onto the glass beads. This corresponded to 10^2^ to 10^3^ cells per gram of glass beads in each microcosm.

After inoculation, we established three hydration regimes in our experiments (Fig. 1C). We established constant hydration conditions throughout the duration of the experiment (12 days) for the “WET” microcosms (−0.5 kPa) or “DRY” for other microcosms (−6 kPa). The third hydration regime was dynamic (“DYN”) and consisted of two cycles of drying and rewetting induced by varying the height of the liquid column to induce changes in matric potential values (which typically takes a few minutes), as follows: −0.5 kPa (2 days), −3 kPa (2 days), and −6 kPa (2 days), and then the cycle was repeated once (Fig. 1C). Thus, the representative bacterial community was exposed to a combination of two nutrient conditions (high and low), three hydration conditions (WET, DRY, and DYN), and (destructive) sampling performed at six time points (2, 4, 6, 8, 10, and 12 days) for dynamic drying-rewetting events and at three time points (2, 6, and 12 days) for constant hydration conditions. The microcosms were incubated at constant temperature (25°C). We used four microcosm replicates per condition, yielding 48 distinct experimental samples for the dynamic drying-rewetting experiment and 24 samples for each of the constant hydration conditions (dry and wet), totaling 96 samples plus the initial community used for inoculation (undiluted to ensure sufficient DNA yield). For each sample, the entire glass bead matrix of the microcosm was removed and stored at −80°C until further analysis.

### Total nucleic acid extraction and quantification.

Immediately prior to DNA extraction, microcosms were spiked with a plasmid containing the archaeal 16S rRNA gene amplicon to control for DNA loss during the extraction process (see below). Total nucleic acid (NA) extraction was carried out as described previously (19). In brief, the entire glass bead matrix was removed from each microcosm with phosphate buffer and placed in a Lysing Matrix E tube (MP Biomedicals, Heidelberg, Germany). After the addition of a 10% SDS solution and phenol, cells were lysed in a bead mill homogenizer (Bead Ruptor; Omni International, Kennesaw, GA, USA), and the procedure was repeated once using fresh buffer and phenol. The supernatant was collected in phase-lock tubes (Quantabio, Beverly, MA, USA) and further purified first with a phenol-chloroform-isoamyl alcohol solution and then a chloroform-isoamyl alcohol solution in DNA LoBind tubes (Eppendorf, Hamburg, Germany). NA was precipitated overnight at 4°C after the addition of 20% polyethylene glycol and 5 μg of glycogen, followed by a 60-min centrifugation step at 4°C and 16,000 × *g*. The pellet was washed with ice-cold 75% ethanol, dried, and resuspended in 50 μl DNA suspension buffer (10 mM Tris, 0.1 mM EDTA [pH 8.0]). For qPCR, total RNA was removed, and samples were further purified as follows. An aliquot of 10 μl (15 μl for samples with low DNA content) of NA solution was treated with 40 μg/ml RNase A (Promega, Madison, WI, USA) for 15 min at 37°C in a final reaction volume of 50 μl and subsequently column purified with the NucleoSpin gDNA cleanup kit (Macherey-Nagel, Düren, Germany). The final DNA concentration was quantified with the Qubit double-stranded DNA (dsDNA) high-sensitivity (HS) assay kit (Thermo Fisher Scientific) on a microplate reader (Tecan). We retrieved DNA from most porous microcosms (Fig. S1), with only a few exceptions, probably due to failure of target hydration conditions in the microcosm (e.g., loss of liquid connectivity with the medium reservoir and subsequent drying out of the bacterial habitat).

### Internal DNA standard to estimate DNA recovery after extraction.

As an internal standard, we used the full length of the 16S rRNA gene of the archaeon Haloarcula marismortui cloned into pCR2.1-TOPO (Thermo Fisher Scientific). The 16S rRNA gene of archaea is not amplified by the universal primers targeting the 16S rRNA gene of bacteria. After overnight amplification in E. coli, the vector was isolated using a spin miniprep kit (Qiagen, Hilden, Germany). To remove contamination from the host, the extraction was run on a 1.5% agarose gel, and the DNA band corresponding to the plasmid was excised and purified with a commercial kit (Wizard SV gel and PCR clean-up system; Promega). After quantification of DNA using a Qubit assay (Thermo Fisher Scientific), the internal standard DNA was diluted to a final concentration of 0.74 ng/μl. Seven microliters of internal standard was added to each sand microcosm immediately prior to nucleic acid extraction. The extraction efficiency was indirectly quantified through tracking the internal standard. We designed a primer pair targeting a 142-nucleotide (nt) region of the 16S rRNA gene of Haloarcula marismortui used as an internal standard (Table S1) in parallel with the universal primer set accounting for the total community size. A qPCR was run on a Flex Six chip (Fluidigm) in technical triplicates using the same PCR program used for the Fluidigm GE 48.48 chips, and the ratio of the total community size to the spike-in tracer was calculated. Comparisons of amplification DNA signal from the bacterial community and from a DNA spike showed that variations in DNA content among microcosms resulted from variations in bacterial growth rather than from poor efficiency of DNA recovery (Fig. S8).

### Microfluidic quantitative real-time PCR.

Microfluidic qPCR assays were performed using a 48.48 Dynamic Array (Fluidigm Corporation, San Francisco, CA, USA), as described in detail in reference 19. Following the manufacturer’s recommendations, a preamplification step was included in the workflow to provide sufficient copies of the target 16S rRNA gene for the microfluidic reactions, using an universal primer pair amplifying the full length of the 16S rRNA gene (see Table S1). We used 200 nM forward and reverse primers in 1× GoTaq G2 colorless master mix (Promega, Duebendorf, Switzerland) and with 10 μl of purified community DNA from the microcosms (resulting in ≈4 to 30 ng of DNA template) in a final volume of 20 μl. The reactions were run on a PCR thermal cycler (SensoQuest, Göttingen, Germany). After 15 cycles of amplification, an aliquot of 5 μl was treated with 10 U Exonuclease I (Thermo Fisher Scientific) at 37°C for 15 min to remove primers, and as recommended by the manufacturer, the enzyme was heat inactivated at 85°C for 15 min. The rest of the mix was amplified for another 15 cycles, and the product was further visualized on an agarose gel to control for the presence of a single amplicon with size of ≈1.5 kb. The exonuclease-treated amplicons were diluted 5 times in DNA suspension buffer (10 mM Tris [pH 8.0], 0.1 mM EDTA), and 3 μl of this dilution was added to 4.5 μl of sample premix containing qPCR mix (HOT FIREPol EvaGreen qPCR mix Plus [ROX]; Solis Biodyne, Tartu, Estonia) and DNA binding dye (Fluidigm Corporation), giving a final volume of 7.5 μl. After mixing, 5 μl of the sample mix was added to each sample inlet of the Fluidigm Dynamic Array chip. We used species-specific primer pairs that were previously designed and tested for the characterization of our representative soil bacterial community (Table S1). The assay premix was prepared by combining a 20 μM species-specific primer pair with assay loading reagent (Fluidigm Corporation) adjusted to final volume of 7.5 μl with DNA suspension buffer, and 5 μl was added to each assay inlet. Each community DNA sample was run in four technical replicates using the standard qPCR program recommended by the manufacturer (which included a “hot start” step to activate the polymerase and the cycle number increased to 40). A reaction without the DNA template was used as a negative control.

### Data analysis.

To quantify the absolute abundance of each bacterial species, we used a standard curve obtained from genomic DNA templates extracted from pure cultures of each individual species. The input of gDNA was quantified fluorometrically (Qubit high-sensitivity assay for dsDNA; Thermo Fisher Scientific) and combined in equal proportions to prepare a 5-fold dilution series for generating the standard curve. With information on the genome size of each strain used, we calculated a number of genome equivalents per unit mass gDNA for each strain. The threshold cycle (*C_T_*) values were plotted against the calculated number of genome equivalents added as the DNA template (log genome equivalents, Fig. S2), and linear regression was used to calculate the standard curve. Data points in which only one technical replicate (out of 4) returned a *C_T_* value were excluded from further analyses. Calibration samples in which only two technical replicates returned a *C_T_* value were excluded when the standard deviation exceeded 0.5. We used the Grubbs’ test to detect outlier replicate values (with significance level α = 0.05), which were then excluded from the analysis. In total, three independent qPCR arrays were used (chip capacity for 38 samples and 10 inlets for dilution series for the standard calibration plus negative control). The three standard curves showed high reproducibility of slopes but slight differences in *y* axis intersects used to convert *C_T_* values to the number of genome equivalents per sample. For this reason, we used an average of all three curves to account for differences in *y* intercepts (Fig. S2). We computed the goodness of fit (*R*^2^) and the amplification efficiency (derived from the slope) for each curve (Fig. S2 and Table S2). Calibration curves were used to calculate a number of genome equivalents for each strain for all community DNA samples. Measurement values below the theoretical limit of detection (<1 genome in DNA template) were not reported. The size of the bacterial community in each microcosm was back calculated based on the DNA concentration obtained from nucleic acid extraction (quantified fluorometrically with the Qubit dsDNA high-sensitivity assay).

Species absolute abundances (genome equivalents) were log transformed and imported in R (https://www.r-project.org/) for statistical analysis. We used the R package vegan and the function vegdist to calculate Bray-Curtis dissimilarity indices. Subsequently, we used the function heatmap.2 from the R package gplots to produce heatmaps of species abundances and hierarchical clustering with the average method. Phylogenetic clustering of the data was based on neighbor joining of 16S rRNA sequences from the 11 species obtained from the SILVA database (44).

## Supplementary Material

Supplemental file 1
