# Supplementary material for: Rapid Shifts in Bacterial Community Assembly under Static and Dynamic Hydration Conditions in Porous Media
Source: Appl Environ Microbiol. 2019 Dec 13;86(1):e02057-19. doi: 10.1128/AEM.02057-19 (PMC6912082; doi:10.1128/AEM.02057-19)
Supplement: Supplemental file 1 [file AEM.02057-19-s0001.pdf]

Supplementary Material

Title: Rapid shifts in bacterial community assembly under static and dynamic hydration conditions in porous media

Kleyer, Hannah[1]; Tecon, Robin[1] and Or, Dani[1]

[1]Soil and Terrestrial Environmental Physics, Department of Environmental Systems Science, Swiss Federal Institute of Technology in Zurich (ETH Zürich), Universitätstrasse 16, 8092 Zürich, Switzerland

Supplementary Material includes:

Tables S1 to S2

Figs. S1 to S8

Supplementary Table S1. Primers used in real-time PCR.

Name	Sequence (5' to 3')	Tm [°C]	CG Content [%]	Length [nt]	Target pos. 16S	Reference
Species-specific primer pairs						
A_chlo F	CAGCTTGCTGGTGGATTA	60.5	50.0	18	118-283	(1)
A_chlo R	CACCATGCGATGATCAGT	61.3	50.0	18		(1)
B_subt F	GACAGATGGGAGCTTGCT	61.1	55.6	18	82-165	(1)
B_subt R	TGTAAGTGGTAGCCGAAGC	61.0	52.6	19		(1)
B_xeno F	AATACATCGGAACGTGTCCT	61.3	45.0	20	128-209	(1)
B_xeno R	TCCTCTCAGACCAGCTACAG	60.3	55.0	20		(1)
E_coli F	GAAGCTTGCTTCTTTGCTG	61.0	47.4	19	73-177	(1)
E_coli R	TTGGTCTTGCGACGTTATG	62.8	47.4	19		(1)
M_lute F	GACATGTTCCCGATCGCC	67.1	61.1	18	79-183	(1)
M_lute R	CCACCATTACGTGCTGGC	65.7	61.1	18		(1)
P_prot F	GTACTTGTACCTGGTGGCG	61.2	57.9	19	153-270	(1)
P_prot R	GTATTAGCGCCCGTTTCC	62.4	55.6	18		(1)
P_sabi F	GAGTTATGATGGAGCTTGCT	59.0	45.0	20	68-201	(1)
P_sabi R	GGTATGCACCAGAAGGTCTT	61.1	50.0	20		(1)
P_stut F	CTTGCTCCATGATTCAGC	60.1	50.0	18	79-157	(1)
P_stut R	ACGTATGCGGTATTAGCGT	60.2	47.4	19		(1)
R_etli F	GTATACTGTTCGGTGGCG	59.1	55.5	18	781-971	This study
R_etli R	GAAGGGAACCCTGCATC	60.7	58.8	17		This study
S_viol F	GAACGATGAACCACTTCGGTG	65.3	50.0	20	64-179	(1)
S_viol R	GATGCCTGCGAGGGTCAGTA	66.2	57.9	19		(1)
X_auto F	GATCTACCCAATGGTACGG	59.5	52.6	19	68-180	(1)
X_auto R	GTTCATCCAATGGCGATA	60.0	44.4	18		(1)
Universal primer pairs						
27F	AGAGTTTGATCCTGGCTCAG	61.5	50	20		(2)
1492R	CGGTTACCTTGTTACGACTT	58.7	45	20		(2)
1099F mod	AACGAGCGCAACCCT	61.2	60	15		modified from (3)
1407R mod	GACGGGCGGTGTGTA	60.9	66.7	15		modified from (2)
Quantification of internal DNA standard						
H. maris F48	TTAGCCATGCTAGTTGCAC	59.1	47.3	19	48-184	This study
H. maris R184	CTAACTCTCTGCACTCCAGC	59.4	55	20		This study

Supplementary Table S2. Slope and intercept values for the three independent calibration curves for each species obtained by microfluidic qPCR.

Primer pairs	Slope 1<br>Y-inter 1	Slope 2<br>Y-inter 2	Slope 3<br>Y-inter 3	Slope AVG<br>Y-inter AVG	Slope StDEV<br>Y-inter StDEV
A. chlorophenolicus	-3.39	-3.27	-3.31	-3.32	0.05
	33.19	31.88	31.37	32.14	0.77
B. subtilis	-3.18	-3.31	-3.42	-3.30	0.10
	27.83	27.14	26.36	27.11	0.60
B. xenovorans	-3.17	-3.20	-3.31	-3.23	0.06
	28.15	26.62	26.50	27.09	0.75
E. coli	-3.23	-3.13	-3.33	-3.23	0.08
	30.08	28.48	31.83	30.13	1.37
M. luteus	-3.17	-3.09	-3.14	-3.13	0.03
	33.39	32.04	31.62	32.35	0.75
P. sabinae	-3.29	-3.17	-3.22	-3.23	0.05
	29.08	27.59	26.29	27.65	1.14
P. protegens	-3.29	-3.40	-3.04	-3.24	0.15
	31.79	34.35	31.82	32.65	1.20
P.stutzeri	-3.09	-2.97	-3.32	-3.13	0.14
	31.15	32.26	32.73	32.04	0.66
R. etli	-3.36	-3.41	-3.40	-3.39	0.02
	29.86	28.77	28.83	29.15	0.50
S. violaceoruber	-3.20	-3.28	-3.20	-3.23	0.04
	29.51	28.36	30.41	29.43	0.84
X. autotrophicus	-3.26	-3.60		-3.43	0.17
	31.64	32.28		31.96	0.32
Universal	-3.39	-3.30	-3.35	-3.35	0.04
	29.07	29.73	27.53	28.77	0.92

Supplementary Figures

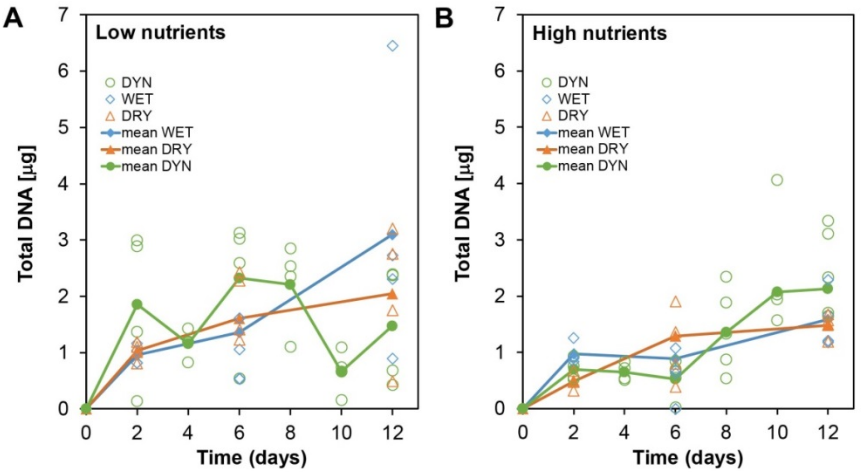

Figure S1. DNA extraction from microcosms. Total nucleic acids extracted from four replicated glass-bead microcosms per time point and treatment (dynamic or static wet and dry hydration conditions), with low (A) or high (B) concentration of nutrients (resp. 0.01× and 0.1× TSBM liquid growth medium). No significant difference in total extracted DNA was observed based on hydration regimes or low or high nutrient concentration. Concentration of total DNA was quantified with the Qubit fluorometric assay specific for detection of double-stranded DNA.

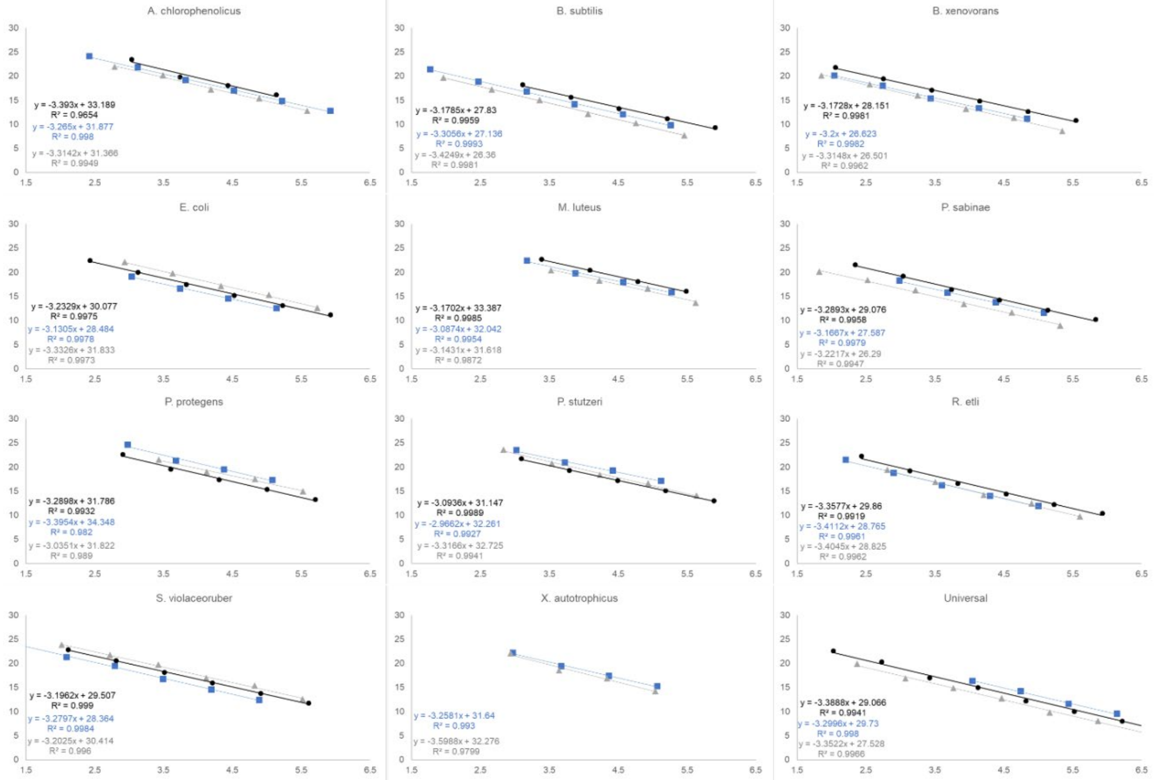

Figure S2. Standard calibration curves. Standard calibration curves for target bacterial species used in the microfluidic assay. Curves were constructed from fivefold serial dilutions of a mixture of purified genomic DNA from eleven individual species (Table 1) pooled in equal amounts and run in parallel reaction with each individual species-specific primer pair or with a universal primer pair (Table S1). Based on genomic DNA concentrations in the stock solution and on the genome size the number of genome equivalent copies was calculated and plotted against the cycle threshold for each species-specific qPCR assay (see Table S2 for details). Data evaluation was performed with the Fluidigm software from four technical replicates per dilution. Data points for average value from four replicates. For each of the three qPCR-chips a standard calibration was calculated, displayed in black, blue and grey. Equations of fitted linear regression lines and R2 values are shown, calculated from average Cq values. For data evaluation the average values from all three calibrations were used.

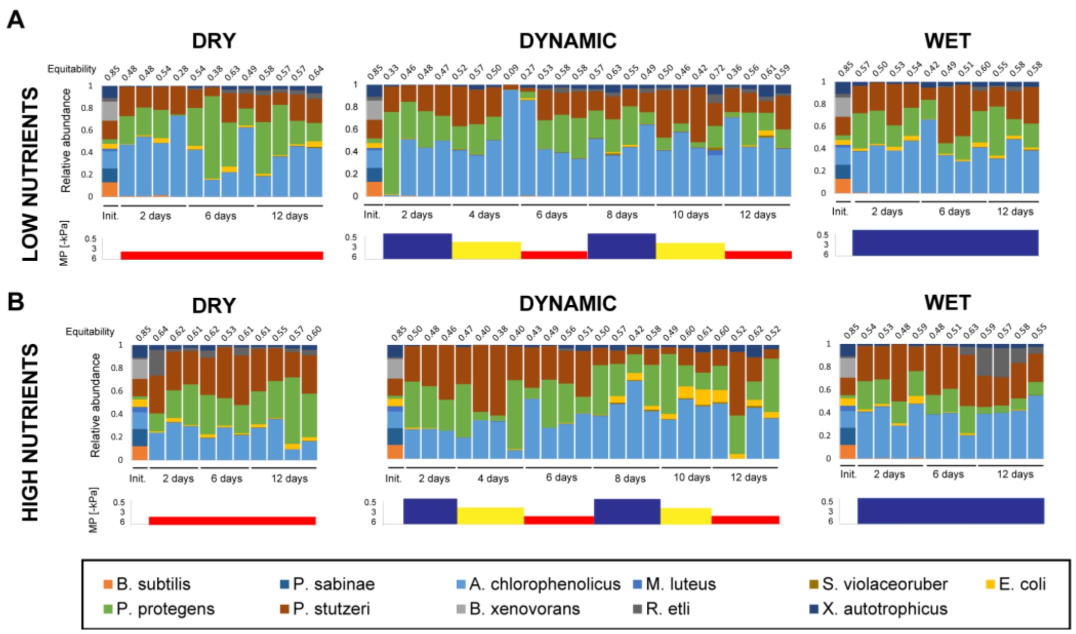

Figure S3. **Bacterial community composition in hydration-controlled microcosms.** The synthetic bacterial community was exposed to dynamic drying-rewetting cycles and compared to static wet or dry conditions under low (A) or high (B) nutrient conditions (resp. 0.01× and 0.1× TSBM liquid growth medium) for a maximal period of 12 days post inoculation. Absolute abundances were calculated for each species based on the absolute number of genome equivalents detected in each microcosm. Species relative abundance and community evenness (Shannon's equitability index) are shown for each sampling day and four replicate microcosms (when only three replicates are shown, it indicates that the fourth replicate produced insufficient amounts of DNA for analysis). Matric potential (MP) histograms recapitulate the changes in hydration conditions from relatively dry (-6 kPa) to relatively wet (-0.5 kPa).

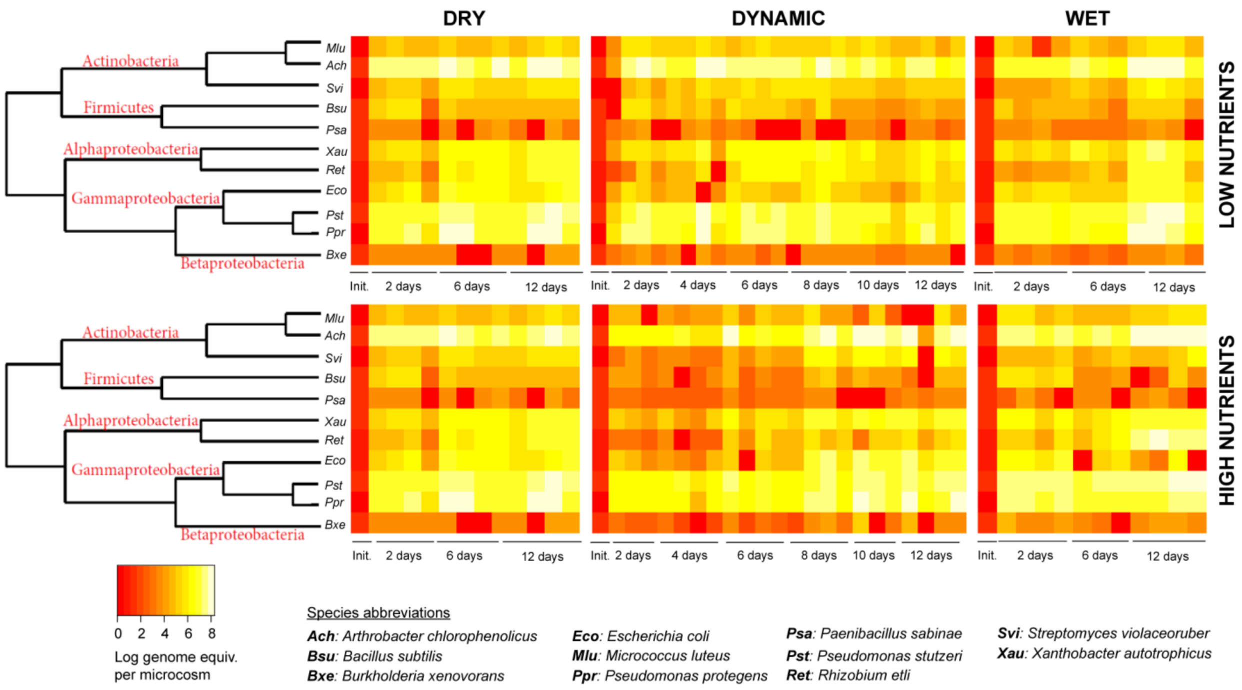

Figure S4. **Species clustering based on phylogeny.** Heatmaps show the species absolute abundances (log-transformed) calculated based on the absolute number of genome equivalents detected in each microcosm. The initial inoculum ('Init.') and 3-4 independent replicate microcosms per treatment and time point are shown. Individual species were clustered on a phylogenetic tree based on the sequence of the 16S rRNA gene.

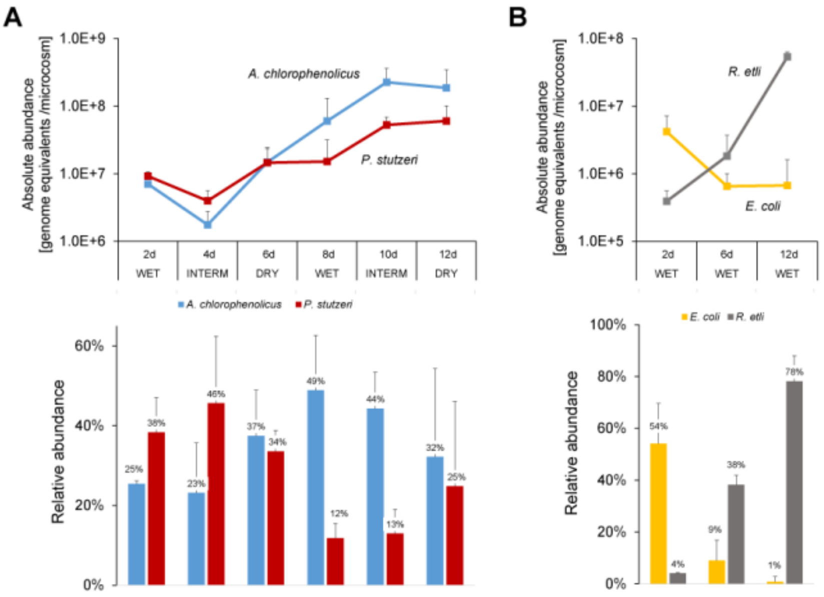

Figure S5. Information on species absolute abundance explains changes in relative abundance patterns. (A) Absolute and relative abundance development superimposed for a subset of two dominant species, *P. stutzeri* and *A. chlorophenolicus.* (data from DYN conditions with high nutrient concentration). Time (in days post inoculation) and hydration conditions are indicated. (B) Relative and absolute abundance development superimposed for a subset of two species with lower abundance levels, *E. coli* and *R. etli* (data from WET conditions with high nutrient concentration). All panels show average values calculated from 3-4 replicate microcosms. Error bars are one standard deviation.

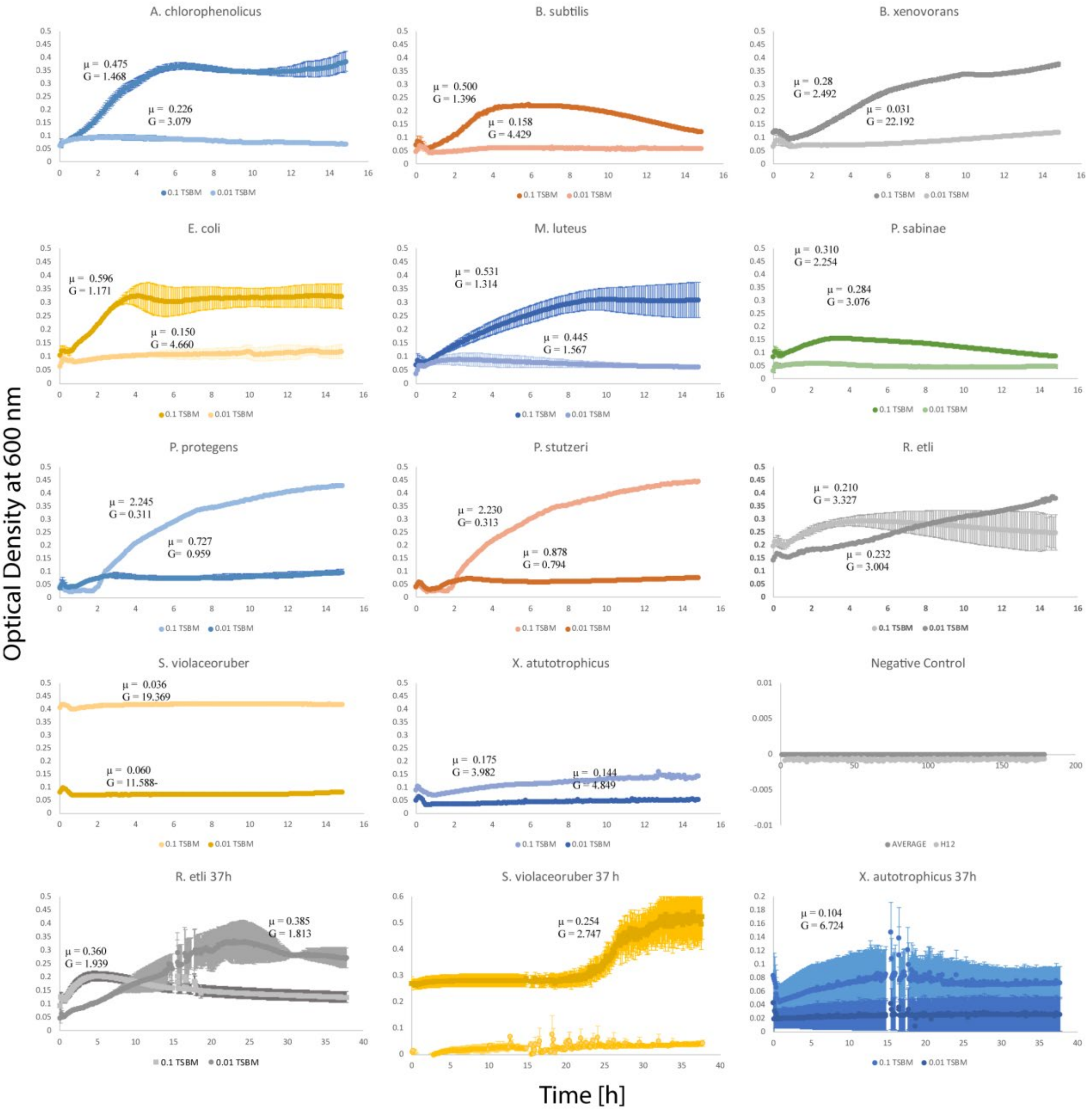

Figure S6. **Bacterial species growth kinetics in batch culture.** We tested growth of each individual bacterial species under high nutrient conditions (0.1x TSBM medium) and low nutrient conditions (0.01x TSBM medium). Bacterial cultures from plate were resuspended in respective medium, diluted to an OD of 0.1 and incubated in a 96-well plate at room temperature. Growth was measured in a plate reader (Tecan, Männedorf, Switzerland) as absorbance at 600 nm, A600 every 5 minutes over a total of 21 hours. Cultures were mixed by orbital shaking for 15 s at 260 rpm before each measurement. Background was subtracted before further analysis of growth data. Growth curves for high nutrient conditions are shown in saturated colour (e.g. blue) while the low nutrient conditions is shown in same colour but lighter tone (e.g. light blue).

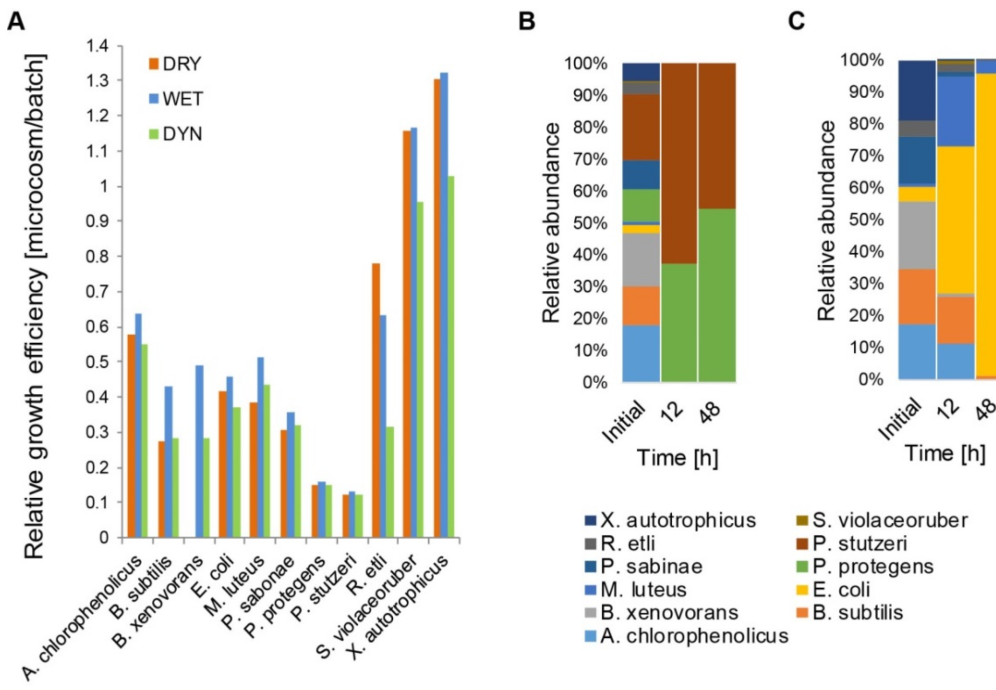

Figure S7. Comparisons of growth in microcosms and batch cultures. (A) Ratio of generation time measured in microcosm and shaken liquid (batch) culture. All species grow faster in batch than in glass beads microcosms (ratio <1), with the exception of *S. violaceoruber* and *X. autotrophicus*. (B, C) We modelled changes in bacterial community composition over time based on specific growth rates measurements in individual batch cultures for each species, with the same initial community composition than in the microcosms experiment. The community was rapidly (after < 3 h) dominated by *Pseudomonas protegens* and *Pseudomonas stutzeri* (B). When the two *Pseudomonas* species were removed from the analysis (C), the community became almost entirely dominated by *E. coli* after 48 h.

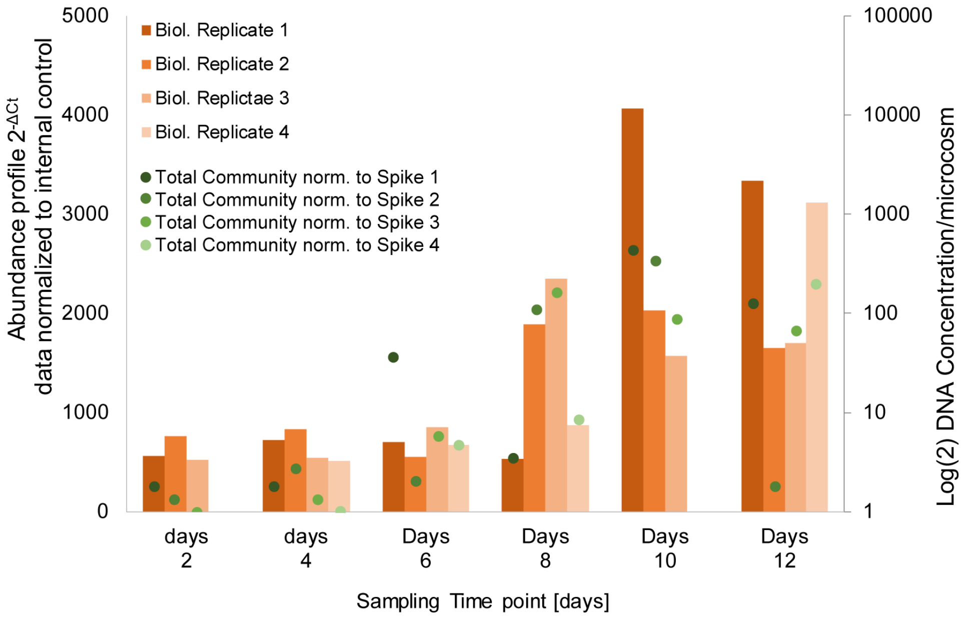

Figure S8. **Evaluation of community size normalized to internal DNA standard to assess DNA extraction efficiency.** Comparison of DNA concentration after extraction (red bars) and total community count obtained from the universal primer pair, normalized to the internal DNA standard (green dots). DNA concentration was quantified in a fluorometric assay specific for double stranded DNA (Qubit High Sensitivity assay for dsDNA assay, Thermo Fisher Scientific) and compared to computed fold-change increase of universal primer set compared to the DNA standard used to spike each sample prior to total NA-extraction. Variations in total extracted DNA between the four biological replicates and between sampling time points are reflected in fold-change increase of total community normalized to the internal DNA standard, suggesting that extraction efficiency is comparable among replicates and sampling time points.

References

1. H. Kleyer, R. Tecon, D. Or, Resolving Species Level Changes in a Representative Soil Bacterial Community Using Microfluidic Quantitative PCR. *Front Microbiol* **8**, 2017 (2017).
2. D. Lane, 16S/23S rRNA sequencing. Nucleic acid techniques in bacterial systematics., 115-175 (1991).
3. S. E. Dyksterhouse *et al.*, Cycloclasticus pugetii gen. nov., sp. nov., an aromatic hydrocarbon-degrading bacterium from marine sediments. **45**, 116-123 (1995).
